# Editorial: Bottom-Up Approach: A Route for Effective Multi-Modal Imaging of Tumors

**DOI:** 10.3389/fonc.2021.812472

**Published:** 2022-01-14

**Authors:** Ruoxi Xie, Changqiang Wu, Lu Yang, Peng Mi, Dong-Hyun Kim, Min Wu

**Affiliations:** ^1^ Huaxi Magnetic Resonance Research Center (HMRRC), Department of Radiology, Functional and Molecular Imaging Key Laboratory of Sichuan Province, West China Hospital, Sichuan University, Chengdu, China; ^2^ Medical Imaging Key Laboratory of Sichuan Province and School of Medical Imaging, North Sichuan Medical College, Nanchong, China; ^3^ The Department of Urology, The Institute of Urology, West China Hospital, Sichuan University, Chengdu, China; ^4^ Department of Radiology, Center for Medical Imaging, and State Key Laboratory of Biotherapy, West China Hospital, Sichuan University, Chengdu, China; ^5^ Department of Radiology, Feinberg School of Medicine, Northwestern University, Chicago, IL, United States

**Keywords:** tumor, multi-modal probes, multi-parametric imaging, MRI, CT, fluorescence, PET-CT, PET-MRI

## Preface

With the development of multidisciplinary integration, multi-modal imaging has become an important concept that is very popular in relevant research fields and has expanded to accommodate a wide range of advanced technologies. This change is something we are happy to see because it brings more possibilities, which further provide more perspectives and information for cancer research. This Research Topic gathers information about various technologies or methodologies, to consider the advantages of each and finally provide solutions for cancer diagnosis and treatment.

## Current Status and Innovation of Multimodal Imaging

Imaging has become an indispensable means in the process of tumor diagnosis and clinically and the wide range of available imaging modalities, some of which are described herein, provide strong tools in discovering information about cancer. Among these available imaging modalities, magnetic resonance imaging (MRI), is a commonly used tool in clinical settings and is usually one of the first choices for tumor diagnosis. It has superior soft-tissue resolution to any other imaging modality, which makes it suitable for the diagnosis of most tumors. In addition, enhanced MRI detection can further strengthen its application. However, the relatively long scanning time and various types of artifacts sometimes limit its function. From the perspective of the articles in this Research Topic, exploiting advanced MRI sequences for better imaging effect is still necessary for tumor diagnosis.

Yuan et al. explore the diagnostic efficacy of the combination of different MRI sequences, including T_2_-weighted spectral attenuated inversion recovery (SPAIR) imaging, dynamic contrast-enhanced (DCE) imaging, and diffusion-weighted (DW) imaging. They found that T_2_ SPAIR and DCE plus DW imaging provided useful information for evaluating T staging and grading in bladder cancer. As a multi-parametric imaging modality, MRI can give far more information than we have ever thought. Zheng et al. found that the correlation between the parameters of intravoxel incoherent motion diffusion-weighted imaging (IVIM-DWI) and the expression of Ang-2 and TKT to be very useful for developing personalized treatment plans by non-invasive evaluation. Liu et al. examine the mono-exponential IVIM model. They outline that it is superior to the bi-exponential IVIM model in differentiating the pathological grades of esophageal squamous cell carcinoma (ESCC), proving it to be an ideal imaging modality for predicting pathological grades of ESCC.

Computed tomography (CT) is another commonly used imaging modality. Compared with MRI, it does not have an impressive soft tissue resolution, but the quicker scanning time for better temporal resolution and relatively low cost make it still a necessary inspection method for cancer diagnosis. Enhanced CT scanning is particularly sensitive to some tumors such as hepatoma and some abdominal tumors. Consequently, improving the performance of the CT devices and developing CT imaging agents with higher contrast and specificity are potential approaches to increasing the accuracy of CT in tumor diagnosis. Furthermore, similar to other imaging modalities, the radiomics model based on CT can excavate more information from a large number of images and effectively provide a prediction for the development of cancer.

Ultrasound (US) is an imaging modality with relatively high penetration that has the capability of real-time imaging. Based on contrast enhanced US, Tang et al. used a new index named time difference of arrival (TDOA), which uses the time difference between the contrast agent’s arrival time of the lesion and adjacent lung tissue to discriminate the pathologic types of peripheral pulmonary lesions (PPLs). This retrospective study found that TDOA significantly distinguished benign inflammation and malignant PPLs, which increased the diagnostic accuracy of CEUS. However, the properties of sounds restrict its performance when meeting the tumors surrounded by air or bones. Fortunately, some advanced US contrast agents and invasive US scanning methods can help solve these problems (1). Nuclear imaging relies on radioactive rays emitted by radionuclides and sometimes shares some characteristics of molecular imaging like cellular or molecular level specificity. More advanced fusion technology in nuclear imaging such as positive electron emission CT (PET/CT) and PET/MRI can simultaneously obtain metabolism information and anatomical structure, providing the precise location and behaviors of tumor development. In this field of concern, the development of new tracers is still very popular. Zhou et al. performed an intra-individual comparison and found that 18F-PSMA-1007 showed superiority over 18F-FDG because of its high detecting rate of PC lesions and excellent tumor uptake. Generally speaking, from this brief review above, it is clear that the commonly used imaging methods have different advantages, but there is still a lot of room for improvement.

Molecular imaging is another important branch of multi-modal imaging, which relies on composite probes integrating different parts with multiple functions to realize precise tissue imaging. The tumor occurrence is a multi-factor process at the cellular level, subcellular level, and molecular level, thus providing a huge stage for the application of molecular imaging. The recent innovation of molecular imaging generally includes aspects such as bimodal imaging, targeting, structure, synthesis, mechanism of drug release. Bimodal imaging requires the probe to be highly integrated and combines the advantages of different imaging modals, thus we can dig out more information with higher efficiency. For instance, Xie et al. reported a bimodal MRI/near infrared fluorescence (NIRF) imaging nanoprobe, ^D^ANG/Cy7-SPIONs (2) that used the retro-enantio isomer of angiopep-2 to mediate the nanoprobe targeting to glioma site. Due to the integration of Cy7 dye and SPIONs core, this nanoprobe finished the enhanced MR imaging and NIRF imaging of glioma with high specificity and sensitivity. The MRI/optical imaging bimodal imaging probe has been applied in many studies (3), because of its combination of high anatomical resolution from MRI, high sensitivity, and real-time imaging property from optical imaging (4).

In terms of the capability of targeting, finding new tumor targets with their corresponding ligands is an effective way to improve the targeting efficiency of the composite probes. Small molecules, like peptides, have become one of the most popular ligands for targeting, because they are small, easy to synthesize, and generally low immunogenic (5). Genomics research also helps in the discovery of more targets to develop new small molecule targeting ligands. Some research designed molecular probes with camouflage containing some intrinsic components in the body to deceive tumor cells. This biomimetic strategy has also achieved some impressive results. Deng et al. produced an aggregation-induced emission (AIE) probe coated with the NK cell membrane, NK@AIEdots, in which the NK cell membrane helped the probe cross the blood-brain barrier and target the glioma and finally realize through skull NIRF imaging and tumor treatment *via* photothermal effect (6). Then, the structure of the whole probe is strongly associated with its mechanism of drug release.

Recently, many composite probes with the activatable design have been developed, as they provide impressive lesion to normal tissue contrast, which is particularly valuable when we want to make a strict distinction between the two, for example during surgery. Zhan et al. designed an enzyme-activatable NIR-II nanoprobe unperturbed by tissue or background (A&MMP@Ag_2_S-AF7P) (7). In the normal state, the nanoprobe did not have effective imaging performance because of the quenching effects of its special structure. The structure of the nanoprobe would be destroyed by the MMP-14, which is an enzyme highly expressed on neuroblastoma, that can cleave to a part of the nanoprobe, resulting in the activation of the nanoprobe to emit significant NIRF imaging signals. As for the synthesis of the probes, a faster and simpler method is required. Therefore, the research seeking a product with faster and simpler synthesis while maintaining a similar efficacy is of particular concern.

In summary, as the potential innovations mentioned above indicate, there is still much room for the research and development of multi-modal imaging from a purely molecular imaging perspective. In the future, these composite probes will achieve incredible accuracy.

Many works in this Research Topic examine artificial intelligence (AI) methodologies such as radiomics, machine learning, and deep learning and apply them to the research of cancer imaging with impressive results, indicating the need to expand the concept of multi-modal imaging. Molecular imaging focuses on the characteristics of tumor changes at the cellular and molecular levels. AI methodology for imaging, a rapidly developing research field, can capture the feature and heterogeneity of the tumor across its whole volume. With certain processing, the extracted information from AI methodologies can be used as indicators for the diagnosis and prognosis of cancer. He et al. built a radiomics feature-based predictive model to predict the occurrence of microvascular invasion (MVI) in patients with hepatocellular carcinoma (HCC). They built the individual predictive nomogram including the Fradiomics score (Rad-score) and existing predictors (alpha fetoprotein, neutrophilic granulocyte, and preoperative hemoglobin) and found that its discrimination efficacy was higher than the existing predictors mentioned above, indicating that the radiomics feature-based predictive model could improve the preoperative prediction of MVI in HCC. Gu et al. developed a prediction model based on two clinical factors, namely the age and CA125 level and four radiological criteria by CT, which are abdominal bowel metastasis, spleen metastasis, diaphragmatic metastasis, and retroperitoneal lymph node enlargement (RLNE). Their results indicate that this model could predict unsatisfactory debulking surgery in patients with advanced ovarian cancer (AOC). Li et al. undertook a retrospective study to verify the efficacy of their radiomics model based on multi-parametric MRI on a diagnosis of prostate cancer (PCa). They found that when this model was added to PI-RADS, the performance of the PI-RADS was significantly improved for PCa diagnosis, suggesting the function of radiomics in improving the diagnostic performance of PI-RADS v2.1 in PCa. These studies prove that after the features in the images are extracted and processed by dimensionality reduction, the analyzed data can change the quality of the information provided by the traditional imaging modalities. Sometimes these features can be connected to other non-imaging features from laboratory indicators or genomics to improve the diagnosis and prognosis of cancer.

These imaging features can be associated with genomic, transcriptomic, and proteomic characteristics (8). From this point, we can build a bridge between molecular imaging and radiomics, because they can share and contribute critical information to each other. Chu et al. developed a model based on radiomics features to evaluate CXCL8 expression and found that the radiomics features reflected by tumor morphology could be influenced by gene expression, while CXCL8 is a gene that can regulate cytokine–cytokine receptor interaction and neutrophil migration pathway. Finally, they built a novel radiomics model that incorporated 12 radiomics features according to CXCL8 expression and tumor stage, which was a reliable approach to predict prognosis in colorectal cancer (CRC) patients. This type of study links the differences in genotypes with the heterogeneity in radiomics and found a correlation between them.

On the one hand, the findings in this Research Topic provide direct information for tumor imaging. On the other, the connection between differences in radiomics or other AI methodologies, as well as differences at the cellular and molecular levels, can also indirectly provide new targets for tumor molecular imaging. Moreover, given the rich information provided by AI methodologies, molecular imaging probes can also be more accurate in time and space, which not only identifies the location of the tumor but also specifically identifies the tumor staging and completes the time-specific imaging. At this level, multi-modal imaging is more like a new technology that integrates multiple imaging methodologies, where we can make full use of the information provided by images, computer science, materials, etc. to comprehensively re-consider the whole process of cancer development.

## Epilogue

We are grateful to the authors for contributing to this collection, providing new ideas and perspectives that have also inspired us deeply. This Research Topic will continue to welcome diverse tumor imaging studies and encourage the integration of different imaging methods. We believe that multi-modal imaging will become one of the ultimate solutions for tumor diagnosis and treatment.

## Author Contributions

RX and MW wrote the paper text. All others were guest associate editor of the Research Topic and edited the text.

## Funding

This work was supported by the Chengdu International Science and Technology Cooperation Funding (Grant 2019-GH02-00074-HZ), the Chengdu Science and Technology Bureau (Grant 2021-YF05-00698-SN), the 1·3·5 Project for Disciplines of Excellence-Clinical Research Incubation Project, West China Hospital, Sichuan University (Grant 2021HXFH035), the Functional and Molecular Imaging Key Laboratory of Sichuan Province (Grant 2012JO0011), and the Scientific and Technological Achievements Transformation Fund of West China Hospital, Sichuan University (Grant CGZH21002).

## Conflict of Interest

The authors declare that the research was conducted in the absence of any commercial or financial relationships that could be construed as a potential conflict of interest.

## Publisher’s Note

All claims expressed in this article are solely those of the authors and do not necessarily represent those of their affiliated organizations, or those of the publisher, the editors and the reviewers. Any product that may be evaluated in this article, or claim that may be made by its manufacturer, is not guaranteed or endorsed by the publisher.
